# Differing effects of beta-blockers on long-term clinical outcomes following percutaneous coronary intervention between patients with mid-range and reduced left ventricular ejection fraction

**DOI:** 10.1186/s12872-021-01850-9

**Published:** 2021-01-15

**Authors:** Jun Shitara, Ryo Naito, Takatoshi Kasai, Hirohisa Endo, Hideki Wada, Shinichiro Doi, Hirokazu Konishi, Shuta Tsuboi, Manabu Ogita, Tomotaka Dohi, Shinya Okazaki, Katsumi Miyauchi, Hiroyuki Daida

**Affiliations:** 1 Department of Cardiovascular Medicine, Juntendo University Graduate School of Medicine, 2-1-1 Hongo, Bunkyo-ku, Tokyo, 113-8421 Japan; 2 Cardiovascular Respiratory Sleep Medicine, Juntendo University Graduate School of Medicine, 2-1-1 Hongo, Bunkyo-ku, Tokyo, 113-8421 Japan; 3 Sleep and Sleep Disordered Breathing Center, Juntendo University Hospital, 2-1-1 Hongo, Bunkyo-ku, Tokyo, 113-8421 Japan; 4 Department of Cardiology, Juntendo University Shizuoka Hospital, 1129 Nagaoka, Izunokuni, Shizuoka 410-2295 Japan

**Keywords:** Beta-blocker, Ischemic heart disease, Mid-range ejection fraction, Reduced ejection fraction

## Abstract

**Background:**

The aim of this study was to determine the difference in effects of beta-blockers on long-term clinical outcomes between ischemic heart disease (IHD) patients with mid-range ejection fraction (mrEF) and those with reduced ejection fraction (rEF).

**Methods:**

Data were assessed of 3508 consecutive IHD patients who underwent percutaneous coronary intervention (PCI) between 1997 and 2011. Among them, 316 patients with mrEF (EF = 40–49%) and 201 patients with rEF (EF < 40%) were identified. They were assigned to groups according to users and non-users of beta-blockers and effects of beta-blockers were assessed between mrEF and rEF patients, separately. The primary outcome was a composite of all-cause death and non-fatal acute coronary syndrome.

**Results:**

The median follow-up period was 5.5 years in mrEF patients and 4.3 years in rEF patients. Cumulative event-free survival was significantly lower in the group with beta-blockers than in the group without beta-blockers in rEF (*p* = 0.003), whereas no difference was observed in mrEF (*p* = 0.137) between those with and without beta-blockers. In the multivariate analysis, use of beta-blockers was associated with reduction in clinical outcomes in patients with rEF (hazard ratio (HR), 0.59; 95% confidence interval (CI), 0.36–0.97; *p* = 0.036), whereas no association was observed among those with mrEF (HR 0.74; 95% CI 0.49–1.10; *p* = 0.137).

**Conclusions:**

Our observational study showed that use of beta-blockers was not associated with long-term clinical outcomes in IHD patients with mrEF, whereas a significant association was observed in those with rEF.

## Background

Ischemic heart disease (IHD) is the most common cause of heart failure (HF) with left ventricular (LV) dysfunction [1–3]. The severity of LV systolic dysfunction is an important prognostic factor in patients with IHD [4], possibly because impaired LV systolic function increases likelihood of developing HF and ventricular arrhythmia, both of which could be fatal conditions. In the last few decades, significant advancements in treatments for IHD have been made through the adoption of healthy behaviors (i.e., restriction of salt intake, endorsement of physical activity, smoking cessation), improvement in interventional cardiology (i.e., less invasiveness in devices for catheter intervention, development of coronary stents) and evidence-based medical therapy, including beta-blockers, angiotensin-converting enzyme inhibitors (ACEIs), statins, and aspirin [5]. Despite the advancements, cardiovascular events, such as acute coronary syndrome (ACS), development of HF, and sudden cardiac death, still remain a matter of concern for patients with IHD, which requires further refinement of treatment strategies in order to reduce subsequent cardiovascular events in those patients. Beta-blockers have been established as evidence-based medical therapy to prevent secondary cardiovascular events [6, 7]. However, the evidence has originated largely from studies examining patients with myocardial infarction, especially those complicated with HF or LV systolic dysfunction. Furthermore, there has been debate over the long-term beneficial effects of beta-blockers in IHD patients without HF or previous myocardial infarction. It is possible that the effects of beta-blockers may vary depending on LV ejection fraction (EF), considering the inconsistent results between studies in patients with and without reduced EF (rEF). Recently, the European Society of Cardiology guideline has proposed a new classification for HF which defines those with EF of between 40 and 49% as mid-range EF (mrEF), for those who have not been classified as either HF with rEF or HF with preserved EF. To date, optimal medical therapy for IHD patients with mrEF has not been established and few studies have examined to see if beta-blockers would be useful in those with mrEF. We hypothesized that effects of beta-blockers in IHD patients would be different between patients with mrEF and those with rEF. In this study, we aimed to investigate differences in the effects of beta-blockers on long-term clinical outcomes in IHD patients with mrEF and those with rEF.

## Methods

### Subjects

We used data from an observational cohort which consists of 3508 consecutive IHD patients who underwent their first percutaneous coronary intervention (PCI) at Juntendo University Hospital (Tokyo, Japan) between January 1997 and October 2011. Three hundred and sixty-three patients whose information on EF were missing, were excluded from analyses. Among 3145 patients, we identified individuals with EF < 50% at the time of their PCI and they were subsequently grouped into two groups according to ranges of their EF. Patients with EF of between 40 and 49% were classified as mrEF group, while those with EF < 40% were classified as rEF group. For each group, we further divided the study population into two groups according to whether they were prescribed with beta-blockers or not at discharge. Decisions on the prescription of beta-blockers were at the discretion of attending doctors based on patients’ clinical status (i.e., comorbid with hypertension, presentation of acute myocardial infarction as the type of IHD, presence of ventricular arrhythmia). Associations between beta-blocker and risks of clinical events were assessed in patients with rEF and mrEF groups, separately.

Informed consent was obtained from all patients before performing PCI. This study was conducted under the approval of our institutional review board in accordance with the Declaration of Helsinki. The ethics application approval number was 17-206.

### Data collection

Baseline data, including age, sex, body mass index (BMI), blood pressure (BP), smoking status, family history of IHD, medication use, and comorbidities that include hypertension, diabetes mellitus (DM), dyslipidemia, and chronic kidney disease (CKD), were prospectively collected. In elective cases, blood samples were collected early in the morning of the day of PCI after an overnight fast. Hypertension was defined as systolic blood pressure (BP) ≥ 140 mm Hg, diastolic BP ≥ 90 mm Hg, or medication with antihypertensive drugs. DM was defined as fasting plasma glycemic levels ≥ 126 mg/dL, medication with oral hypoglycemic drugs, or insulin injections. A current smoker was defined as a person who smoked at the time of PCI or who had quit smoking within a year before their PCI. CKD was defined as an estimated glomerular filtration rate (eGFR) of < 60 mL/min/1.73 m^2^, which was calculated based on the Modification of Diet in Renal Disease Study equation modified with a Japanese coefficient using baseline serum creatinine [8].

### Outcomes

The follow-up period ended on December 31, 2011. Survival data and information on clinical events were collected through serial contact with the patients or their families and, for patients who died or underwent follow-up at our hospital, assessed based on medical records. Details of hospital admission and cause of death were supplied by other hospitals or clinics where the patients had been admitted. Investigators performed blinded collection of all data.

In this study, the primary outcome was a composite event of all-cause death and non-fatal acute coronary syndrome (ACS). We defined ACS as ST-elevation myocardial infarction (STEMI), non-STEMI, or unstable angina pectoris. We determined STEMI based on symptoms of ischemia with ST-segment elevation in electrocardiogram and increased serum levels of cardiac enzymes (troponin, creatinine kinase (CK) MB, CK ≥ two-fold increase) [9, 10], and non-STEMI based on symptoms of ischemia without ST-segment elevation in electrocardiogram and increased serum levels of cardiac enzymes. Unstable angina pectoris was determined based on symptoms of ischemia at rest or the presence of a crescendo of symptoms or new-onset symptoms associated with transient ischemic ST-segment shifts and normal serum levels of cardiac enzymes [11].

### Statistical analysis

Results are expressed as means ± standard deviation or median (interquartile range (IQR)) for continuous variables and as ratios (%) for categorical variables. Baseline data were compared using an unpaired *t-*test or Mann–Whitney U test for continuous variables and the chi-squared test or Fisher’s exact test for categorical variables. Kaplan–Meier survival curves were constructed to compare cumulative event rates between mrEF and rEF groups with the log-rank test as a significance test. Cox proportional hazard regression analyses were conducted to identify whether use of beta-blockers would be associated with the primary composite outcome. Factors associated with outcomes were determined using univariate Cox proportional hazard regression analyses with the following variables: age, sex, hypertension, DM, CKD, family history of IHD, current smoking status, low-density lipoprotein cholesterol (LDL-C), high-density lipoprotein cholesterol (HDL-C), LVEF, and presentation of ACS as well as medication use that included statins, ACEIs or angiotensin receptor blockers (ARBs), aspirin and beta-blockers or no beta-blockers. Variables with a *p* value < 0.1 in univariate analyses were included in multivariate Cox proportional hazard regression analyses. A *p* value of < 0.05 was considered significant, unless otherwise indicated. All data were analyzed using JMP 10.0 MDSU statistical software (SAS Institute, Cary, NC, USA).

## Results

**Fig. 1:**
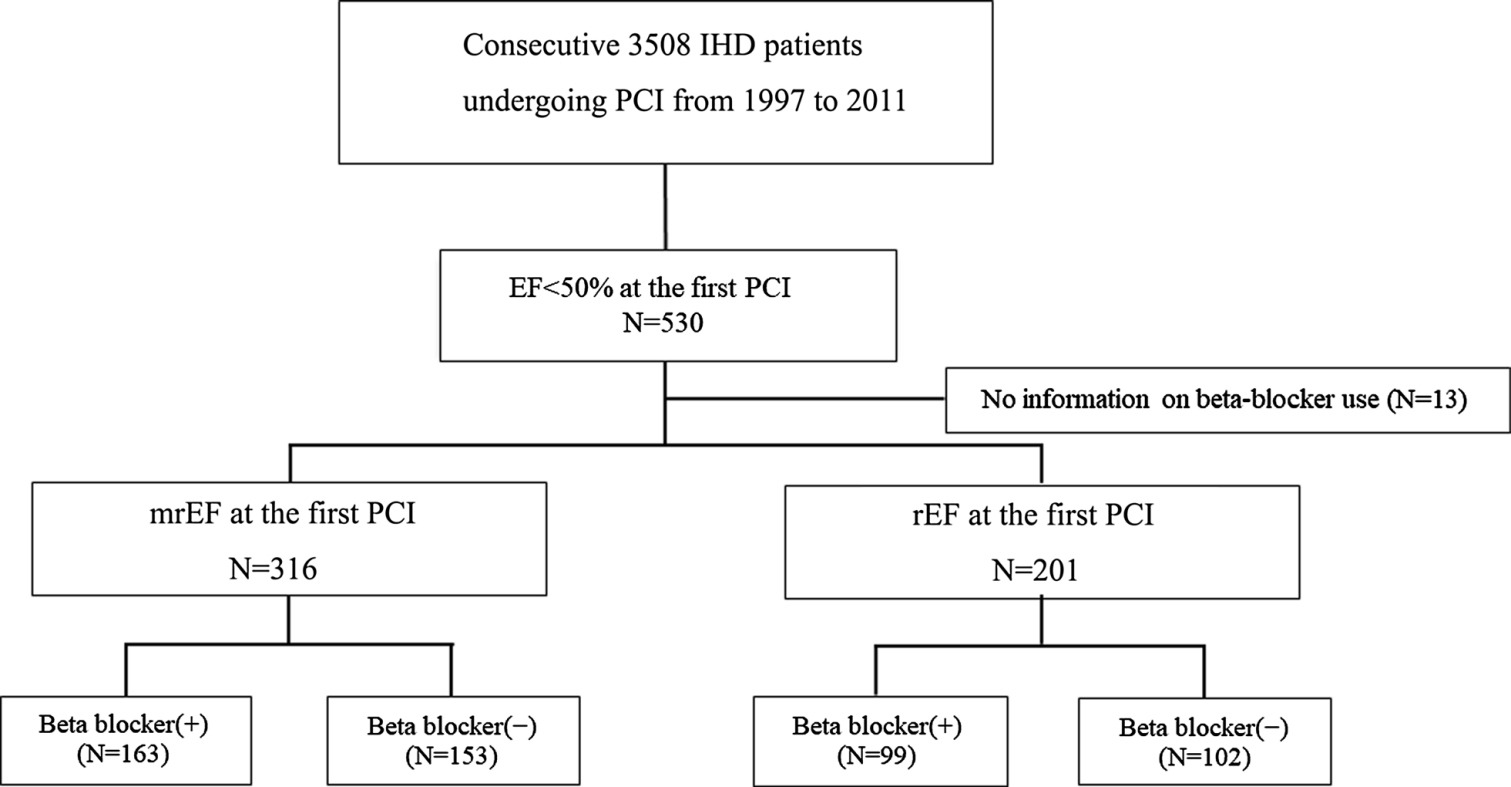
Study flow chart. CAD, coronary artery disease; IHD, ischemic heart disease; mrEF, mid-range ejection fraction; PCI, percutaneous coronary intervention; rEF, reduced ejection fraction

Figure 1 shows a flow chart of the study population. We initially selected 530 patients with LV systolic dysfunction (EF < 50%) among 3508 patients who underwent their first PCI. Patients whose information on prescription of beta-blockers were missing, were excluded (N = 13). In total, 517 patients were enrolled and assigned to two groups: mrEF (EF 40–49%) or rEF (EF < 40%). Both groups of people were subsequently assigned to two groups according to users or non-users of beta-blockers. The prescription rates of beta-blockers were 51.6% and 49.3% in mrEF and rEF, respectively. Table 1 shows the baseline characteristics of each group. In mrEF group, BMI and use of statins were significantly higher in patients with beta-blockers than in those without. In the rEF group, hypertension, diastolic BP and use of aspirin, ACE-Is/ARBs, Type B2/C lesion, drug eluting stent (DES) use, and statins were significantly higher in patients with beta-blockers than in those without. The minimal lumen diameter at baseline was significantly smaller in patients with beta-blockers than in those without.

**Table 1:** Baseline clinical characteristics of the study population

	mrEF			rEF		
	β-blocker ( +) (n = 163)	β-blocker ( −) (n = 153)	*p* value	β-blocker ( +) (n = 99)	β-blocker ( −) (n = 102)	*p* value
Age (years)	64.9 ± 12.1	65.7 ± 10.8	0.567	66.6 ± 10.6	67.8 ± 11.3	0.435
Men (%)	137 (84.1)	125 (81.7)	0.579	84 (84.9)	88 (86.3)	0.774
BMI, (kg/m^2^)	24.1 ± 4.0	23.1 ± 3.5	0.015	24.2 ± 4.0	23.2 ± 4.0	0.082
Hypertension, n (%)	114 (69.9)	96 (62.8)	0.176	76 (76.8)	60 (58.8)	0.007
Diabetes mellitus, n (%)	76 (46.6)	69 (45.1)	0.785	53 (53.5)	51 (50.0)	0.616
CKD, n (%)	58 (36.5)	51 (33.6)	0.589	42 (43.3)	50 (51.0)	0.280
eGFR, (mL/min/1.73 m^2^)	68.7 ± 27.7	66.6 ± 24.9	0.489	63.6 ± 24.6	60.0 ± 38.5	0.427
Family history of IHD, n (%)	47 (29.2)	36 (23.5)	0.255	28 (28.6)	21 (20.6)	0.189
Current smoking, n (%)	43 (26.4)	41 (26.8)	0.933	21 (21.2)	20 (19.6)	0.778
Hemoglobin, g/dL	14.5 ± 1.3	13.2 ± 2.1	0.319	13.2 ± 1.8	12.6 ± 2.1	0.131
LDL-C, mg/dL	111.1 ± 32.3	115.2 ± 31.5	0.249	114.9 ± 38.1	115.0 ± 37.8	0.983
HDL-C, mg/dL	44.8 ± 14.3	44.7 ± 14.6	0.983	41.2 ± 10.7	41.5 ± 12.8	0.867
Triglycerides, mg/dL	131.3 ± 71.6	121.9 ± 74.1	0.251	117.8 ± 58.3	117.5 ± 65.8	0.974
Systolic BP, mmHg	135.6 ± 25.6	132.7 ± 21.9	0.289	124.7 ± 23.1	120.1 ± 21.0	0.143
Diastolic BP, mmHg	74.6 ± 15.3	73.2 ± 13.9	0.401	72.2 ± 13.1	65.8 ± 11.9	< 0.001
LVEF, %	44.5 ± 3.1	44.5 ± 2.9	0.972	30.6 ± 6.8	30.3 ± 7.4	0.755
Multivessel disease, n (%)	99 (61.9)	92 (60.1)	0.752	66 (66.7)	79 (77.5)	0.088
LMT lesion, n (%)	2 (1.2)	7 (4.6)	0.074	6 (6.1)	2 (2.0)	0.137
LAD lesion, n (%)	86 (52.8)	72 (47.1)	0.311	47 (47.5)	52 (51.0)	0.619
MLD at baseline, (mm)	0.3 (0.3–0.4)	0.4 (0.3–0.5)	0.184	0.3 (0.2–0.4)	0.4 (0.3–0.5)	0.036
MLD post-procedure, (mm)	2.7 (2.6–2.8)	2.7 (2.6–2.8)	0.708	2.7 (2.6–2.8)	2.5 (2.3–2.7)	0.062
Reference lumen diameter, (mm)	2.9 (2.8–3.0)	3.0 (2.9–3.1)	0.053	2.9 (2.8–3.0)	3.0 (2.8–3.1)	0.379
Type B2/ C lesion, n (%)	121 (74.2)	102 (66.7.0)	0.140	80 (80.8)	58 (56.9)	< 0.001
Stent size, (mm)	3.0 (3.0–3.25)	3.0 (3.0–3.25)	0.579	3.0 (2.75–3.0)	3.0 (2.75–3.0)	0.780
BMS	86 (52.8)	72 (47.1)	0.311	40 (40.4)	46 (45.1)	0.569
DES	48 (29.5)	46 (30.1)	0.905	37 (37.4)	18 (17.7)	0.003
ACS, n (%)	75 (46.0)	67 (43.8)	0.692	42 (42.4)	47 (46.1)	0.602
*Medications*						
Aspirin, n (%)	153 (93.9)	139 (90.9)	0.312	97 (98.0)	79 (77.5)	< 0.001
Ca-blockers, n (%)	36 (22.1)	34 (22.2)	0.977	19 (19.2)	19 (18.6)	0.919
ACE-Is/ARBs, n (%)	117 (71.8)	104 (68.0)	0.556	70 (70.7)	55 (53.9)	0.014
Statins, n (%)	101 (62.0)	73 (47.7)	0.011	62 (62.6)	36 (35.3)	< 0.001

Data is presented as n (%), mean ± standard deviation, or median (interquartile range)

*ACEI* angiotensin-converting enzyme inhibitors, *ACS* acute coronary syndrome, *ARB* angiotensin receptor blockers, *BMI* body mass index, *BP* blood pressure, *BMS* bare metal stent, *CKD* chronic kidney disease, *DES* drug-eluting stent, *eGFR* estimated glomerular filtration rate, *HDL-C* high-density lipoprotein cholesterol, *IHD* ischemic heart disease, *LAD* left anterior descending artery, *LDL-C* low-density lipoprotein cholesterol, *LMT* left main trunk, *LVEF* left ventricular ejection fraction, *MLD* minimal lumen diameter, *mrEF* mid-range ejection fraction

**Fig. 2:**
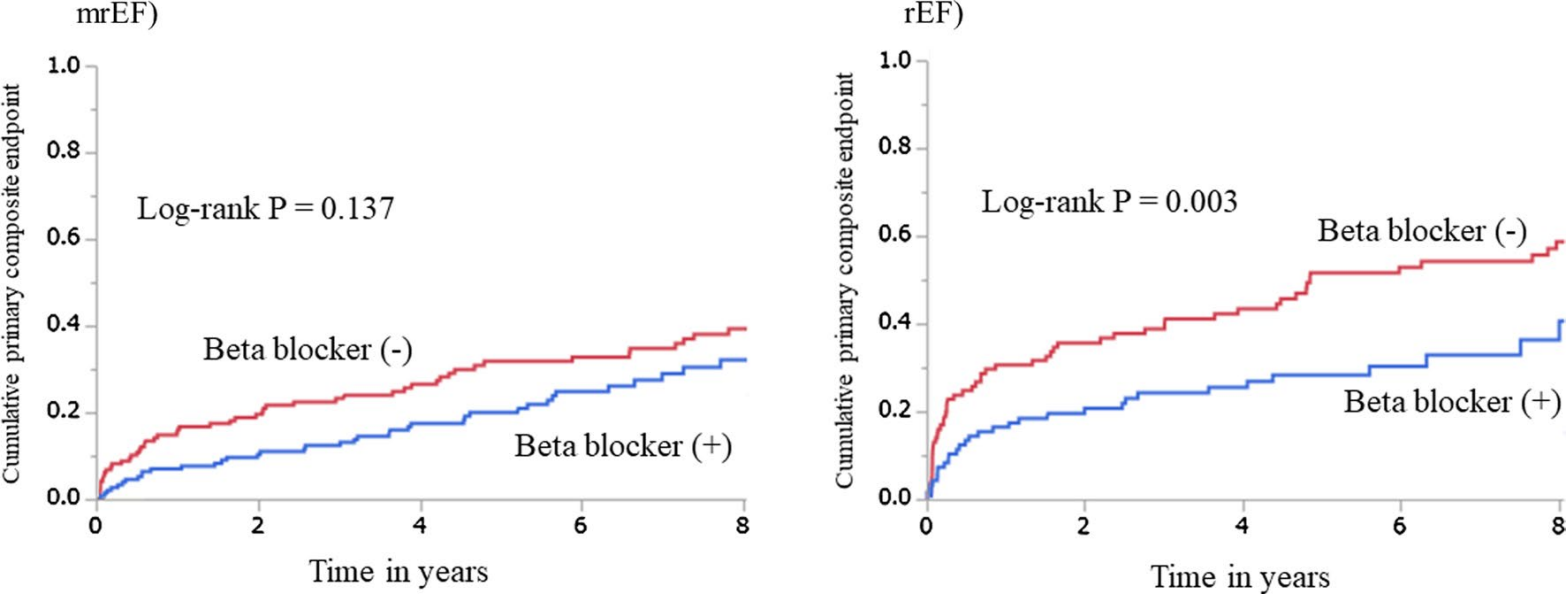
Cumulative incidence rates of the primary composite outcome for those with and without beta-blockers in the mrEF and rEF. There was no significant difference in the cumulative incidence rates of the primary outcome between the two groups in the mrEF (log-rank test, *p* = 0.137). There was a significant difference in the cumulative incidence rates of the primary outcome between the two groups (log-rank test, *p* = 0.003) in the rEF

**Fig. 3:**
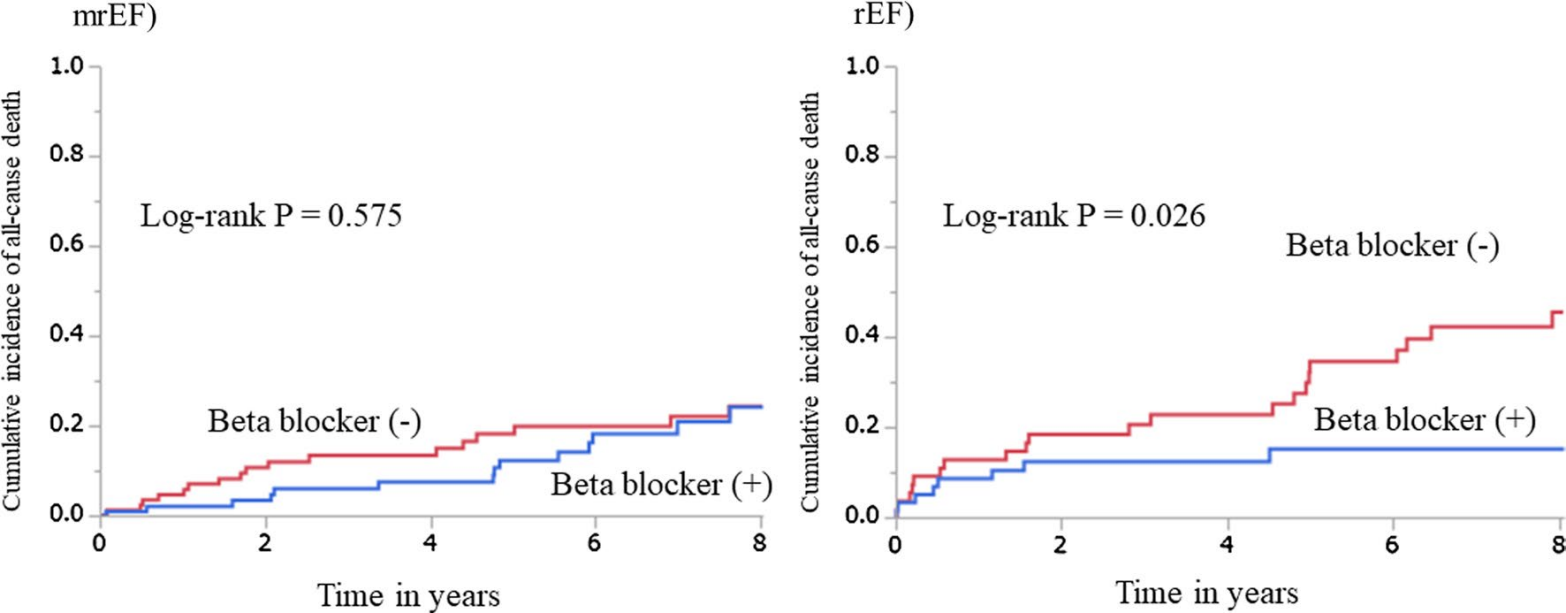
Cumulative incidence rates of all-cause death for those with and without beta blockers in the mrEF and rEF. There was a no significant difference in the cumulative incidence rates of all-cause death between the two groups in the mrEF (log-rank test, *p* = 0.575). There was a significant difference in the cumulative incidence rate of all-cause death between the two groups in rEF (log-rank test, *p* = 0.026)

The median follow-up period was 5.5 (IQR 2.5–9.0) years in the mrEF group and 4.3 (IQR 1.1–7.9) years in the rEF group, and outcome data were fully documented during the entire follow-up period. Figure 2 shows cumulative event rates comparing those with and without beta-blockers. No difference was observed in the incidence of the primary composite outcome between patients with and without beta-blockers in the mrEF group (log-rank test, *p* = 0.137). On the other hand, the cumulative incidence was lower in patients with beta-blockers than those without in the rEF group (log-rank test, *p* = 0.003). The numbers and percentages of each event are shown in Table 2. Similarly, Fig. 3 shows no difference in the cumulative incidence of all-cause death between those with and without beta-blockers in the mrEF group, whereas in the rEF group, the cumulative incidence of all-cause death in patients with beta-blockers was lower than that in those without them. Table 3 shows univariate and multivariate Cox proportional hazards regression analyses including variables with *p* < 0.1 in the univariate analysis for the mrEF group. Table 4 shows univariate and multivariate Cox proportional hazards regression analysis including variables with *p* < 0.1 in univariate analysis for the rEF group similarly. In the multivariate Cox proportional hazards regression analysis for the primary outcome in the mrEF group, greater age, and presence of DM were significant independent predictors of the outcome, while use of beta-blockers was not a significant independent predictor, even in the univariate Cox proportional hazards regression analysis (HR 0.74; 95% CI 0.49–1.10; *p* = 0.137) (Table 3). On the other hand, in the multivariate Cox proportional hazards regression analysis in the rEF group, beta-blocker use was a significant independent predictor of favorable outcomes (HR 0.59; 95% CI 0.36–0.97; *p* = 0.036) in addition to using ACEIs/ARBs. Also, greater age and presence of CKD and ACS were significant independent predictors of unfavorable outcomes. (Table 4).

**Table 2:** Event rates of the composite endpoints

	mrEF		rEF	
	β-blocker ( +) (n = 163)	β-blocker ( −) (n = 153)	β-blocker ( +) (n = 99)	β-blocker ( −) (n = 102)
All-cause death, n (%)	30 (18.4)	30 (19.6)	25 (25.3)	52 (51.0)
Non-fatal ACS, n (%)	13 (8.0)	23 (15.0)	6 (6.1)	9 (8.8)

*ACS* acute coronary syndrome, *mrEF* mid-range ejection fraction, *rEF* reduced ejection fraction

**Table 3:** Results of Cox proportional hazard regression analyses in mrEF

	Univariable			Multivariable		
	HR	95% CI	*p*	HR	95% CI	*p*
Age: *1-year increase*	1.04	1.02–1.06	< 0.001	1.03	1.01–1.05	0.007
Men: *yes*	0.70	0.44–1.17	0.164			
Hypertension: *yes*	1.18	0.78–1.83	0.438			
Diabetes Mellitus: *yes*	1.44	0.97–2.16	0.073	1.56	1.04–2.35	0.033
CKD (eGFR < 60 mL/min/1.73m^2^): *yes*	1.60	1.05–2.41	0.028	1.24	0.80–1.91	0.345
Family History of IHD: *yes*	0.73	0.44–1.18	0.204			
Current smoking: *yes*	0.60	0.35–0.97	0.037	0.81	0.47–1.40	0.435
LDL-C: *1 mg/dL increase*	1.00	0.99–1.00	0.164			
HDL-C: *1 mg/dL increase*	0.99	0.98–1.01	0.258			
LVEF: *1% increase*	0.97	0.91–1.04	0.368			
Multivessel disease: *yes*	1.27	0.84–1.96	0.256			
Acute Coronary Syndrome: *yes*	1.19	0.79–1.77	0.407			
Statins use: *yes*	0.60	0.40–0.90	0.013	0.65	0.43–0.98	0.040
ACEIs/ARBs use: *yes*	1.12	0.73–1.78	0.609			
Aspirin use: *yes*	0.81	0.42–1.82	0.586			
Beta blocker use: *yes*	0.74	0.49–1.10	0.137			

*ACEI* angiotensin-converting enzyme inhibitor, *ARB* angiotensin receptor blocker, *CI* confidence interval, *CKD* chronic kidney disease, *eGFR* estimated glomerular filtration rate, *HDL-C* high-density lipoprotein cholesterol, *HR* hazard ratio, *IHD* ischemic heart disease, *LDL-C* low-density lipoprotein cholesterol, *LVEF* left ventricular ejection fraction, *mrEF* mid-range ejection fraction

**Table 4:** Results of Cox proportional hazard regression analyses in rEF

	Univariable			Multivariable		
	HR	95% CI	*p*	HR	95% CI	*p*
Age: *1-year increase*	1.06	1.04–1.09	< 0.001	1.04	1.02–1.07	< 0.001
Men: *yes*	0.60	0.36–1.05	0.070	0.84	0.48–1.55	0.556
Hypertension: *yes*	0.89	0.58–1.38	0.595			
Diabetes Mellitus: *yes*	0.74	0.49–1.11	0.145			
CKD (eGFR < 60 mL/min/1.73m^2^): *yes*	2.83	1.83–4.46	< 0.001	1.72	1.05–2.86	0.030
Family History of IHD: *yes*	0.93	0.57–1.47	0.759			
Current smoking: *yes*	0.71	0.40–1.19	0.199			
LDL-C: *1 mg/dL increase*	1.00	0.99–1.00	0.589			
HDL-C: *1 mg/dL increase*	1.01	0.99–1.02	0.536			
LVEF: *1% increase*	0.97	0.94–0.99	0.034	0.98	0.95–1.01	0.118
Multivessel disease: *yes*	1.47	0.92–2.43	0.108			
Acute Coronary Syndrome: *yes*	1.90	1.26–2.91	0.002	1.61	1.02–2.54	0.041
Statins use: *yes*	0.55	0.35–0.84	0.005	1.01	0.62–1.65	0.953
ACEIs/ARBs use: *yes*	0.43	0.28–0.64	< 0.001	0.59	0.37–0.95	0.029
Aspirin use: *yes*	0.53	0.32–0.95	0.034	0.66	0.37–1.25	0.194
Beta blocker use: *yes*	0.53	0.34–0.81	0.003	0.59	0.36–0.97	0.036

*ACEI* angiotensin-converting enzyme inhibitor, *ARB* angiotensin receptor blocker, *CI* confidence interval, *CKD* chronic kidney disease, *eGFR* estimated glomerular filtration rate, *HDL-C* high-density lipoprotein cholesterol, *HR* hazard ratio, *IHD* ischemic heart disease, *LDL-C* low-density lipoprotein cholesterol, *LVEF* left ventricular ejection fraction; mrEF, mid-range ejection fraction

## Discussion

This observational study demonstrated that beta-blocker use was not significantly associated with a reduction in the composite of all-cause death and non-fatal ACS among those with mrEF. In contrast, use of beta-blockers was associated with reduction in the events among those with rEF. The prescription rates of beta-blockers were 51.6 and 49.3% in IHD patients with mrEF and rEF, respectively. Our study suggested that the effects of beta-blockers on long-term clinical outcomes in IHD patients may differ based on their ranges of LVEF. In particular, these findings may affect daily clinical practice in patients with IHD and remind physicians the importance of measuring LVEF in patients undergoing PCI.

Prior studies have shown that beta-blockers could improve clinical outcomes in IHD patients [6, 7, 12, 13]. As a result, many guidelines have adopted beta-blockers as one of the first-line drugs for patients with recent myocardial infarction in order to improve their clinical courses by preventing subsequent cardiovascular events, including recurrent coronary events, development of HF, ventricular arrhythmia and death [14, 15], which partly support our finding that use of beta-blockers was associated with a reduction in clinical outcomes for IHD patients who underwent PCI. However, most of the previous studies demonstrating the beneficial effects of beta-blockers have focused on patients with impaired LV systolic function or those complicated with HF. Furthermore, the protective effects of beta-blockers have not been well established in a certain subset of patients whose conditions are not complicated with LV dysfunction [16–18]. Considering the inconsistent results regarding the beneficial effects of beta-blockers between patients with rEF [6, 7] and those on preserved EF [16–18], it has been speculated that LVEF has a role of modifying the effects beta-blockers on prevention of cardiovascular events. However, this has not been fully examined. Our study investigated IHD patients with mrEF, which is a relatively new category for differentiating HF patients according to their LVEF. In this study, the prescription rate of beta-blockers in patients with mrEF was higher than in those with rEF. No associations were observed between beta-blockers and the composite of all-cause death and non-fatal ACS in those with mrEF, whereas the associations were significant in those with rEF, suggesting that the beneficial effects of beta-blockers may vary depending on ranges of LVEF. Prior evidence has suggested that patients with HF with preserved EF would not necessarily benefit from beta-blockers as opposed to those with HF with reduced EF [16–18]. Our study further demonstrated that IHD patients with even modest impairment of LVEF may not be proper candidates for beta-blockers in the long run. Optimal medical therapies have not been established for patients with HF with mrEF as well as IHD patients with mrEF.

Beta-blocker is one of the recommended anti-ischemic drugs for improving angina through reduction in myocardial oxygen consumption and increase in the threshold to myocardial ischemia. Although previous studies have demonstrated that beta-blocker therapy is effective in reducing cardiovascular events, including death and recurrent myocardial infarction (MI), in patients with coronary artery disease, most of the studies were conducted prior to the widespread use of reperfusion therapy, which had dramatically changed the clinical practices for the patients, and the beneficial effects of beta-blockers were limited to patients experiencing MI and those with reduced LVEF [6, 7]. On the other hand, the survival benefits of beta-blockers for IHD patients with mrEF remain unclear. Our study showed no associations between beta-blockers and long-term clinical outcomes, including all-cause death and non-fatal ACS, in IHD patients with mrEF, whereas the association was significant in those with rEF. Given the various adverse effects attributed to beta-blockers, such as depression, dizziness, and bradycardia [19, 20], which are likely to exert in elderly people, broad use of beta-blockers should be avoided, and appropriate patient selection should be made based on the presence of angina, history of MI, reduced LVEF, as well as concomitant HF. It should be noted that in IHD patients with mrEF, the presence of DM was an independent predictor of worse clinical outcomes, which is similar to the results of prior studies [21–23]. This should be further investigated in larger scale studies with a consideration of the effects of beta blockers in mrEF patients with and without DM in the IHD population.

Our study had some limitations. First, it was not possible to exclude unmeasured confounders in this observational study setting, although several variables were adjusted in the multivariate Cox regression analyses. For example, information about the dosage of beta-blockers was not available. Heart rate, which may be suggestive of an effect of the beta-blockers effect, was not considered because no information regarding heart rate is available. Their effects on the clinical outcomes remain unknown. Because subjects of the present study are from the IHD patient population, information regarding the management and treatment of HF, such as B-type natriuretic peptide levels, some medications for HF (i.e., aldosterone antagonists and diuretics), and cardiac implantable devices (e.g., implantable cardioverter defibrillator or cardiac resynchronization therapy which can also affect mortality [24]) is unavailable. Second, data on symptoms related to myocardial ischemia were not available for this study. However, associations between beta-blockers and improvement in angina were beyond the scope of this study. Considering the small number of participants from a single institution in this study, further studies involving post-hoc analyses of large-scale multicentric observational studies or prospective randomized controlled trials are needed to better understand the usefulness of beta-blockers in the management of IHD with mrEF.

## Conclusions

Beta-blockers were not associated with a reduction in the long-term clinical outcomes of IHD patients with mrEF, whereas a significant association was observed in those with rEF. Appropriate patient selection should be made based on the presence of angina, history of MI, concomitant HF as well as LVEF.

## Data Availability

The datasets used and/or analysed during the current study are available from the corresponding author on reasonable request. All data generated or analysed during this study are included in this published article.

## Abbreviations

ACEI: angiotensin-converting enzyme inhibitors
ACS: acute coronary syndrome
ARB: angiotensin receptor blockers
BMI: body mass index
BMS: bare metal stent
BP: blood pressure
CI: confidence interval
CK: creatinine kinase
CKD: chronic kidney disease
DES: drug-eluting stent
DM: diabetes mellitus
eGFR: estimated glomerular filtration rate
HDL-C: high-density lipoprotein cholesterol
HF: heart failure
HR: hazard ratio
IHD: ischemic heart disease
LAD: left anterior descending artery
LDL-C: low-density lipoprotein cholesterol
LMT: left main trunk
LVEF: left ventricular ejection fraction
MI: myocardial infarction
MLD: minimal lumen diameter
mrEF: mid-range ejection fraction
PCI: percutaneous coronary intervention
rEF: reduced ejection fraction
STEMI: ST-elevation myocardial infarction
